# Association of *CETP* Gene Variants With Risk for Vascular and Nonvascular Diseases Among Chinese Adults

**DOI:** 10.1001/jamacardio.2017.4177

**Published:** 2017-11-15

**Authors:** Iona Y. Millwood, Derrick A. Bennett, Michael V. Holmes, Ruth Boxall, Yu Guo, Zheng Bian, Ling Yang, Sam Sansome, Yiping Chen, Huaidong Du, Canqing Yu, Alex Hacker, Dermot F. Reilly, Yunlong Tan, Michael R. Hill, Junshi Chen, Richard Peto, Hongbing Shen, Rory Collins, Robert Clarke, Liming Li, Robin G. Walters, Zhengming Chen

**Affiliations:** 1 Medical Research Council Population Health Research Unit, Nuffield Department of Population Health, University of Oxford, Oxford, England; 2 Clinical Trial Service Unit and Epidemiological Studies Unit, Nuffield Department of Population Health, University of Oxford, Oxford, England; 3 National Institute for Health Research Oxford Biomedical Research Centre, Oxford University Hospital, Oxford, England; 4 Chinese Academy of Medical Sciences, Dong Cheng District, Beijing, China; 5 MRL, Merck Sharp & Dohme Corp, Boston, Masschusetts; 6 Department of Epidemiology and Biostatistics, Nanjing Medical University School of Public Health, Nanjing, China; 7 Department of Epidemiology and Biostatistics, Peking University Health Science Centre, Peking University, Beijing, China

## Abstract

**Importance:**

Increasing levels of high-density lipoprotein (HDL) cholesterol through pharmacologic inhibition of cholesteryl ester transfer protein (CETP) is a potentially important strategy for prevention and treatment of cardiovascular disease (CVD).

**Objective:**

To use genetic variants in the *CETP* gene to assess potential risks and benefits of lifelong lower CETP activity on CVD and other outcomes.

**Design, Setting, and Participants:**

This prospective biobank study included 151 217 individuals aged 30 to 79 years who were enrolled from 5 urban and 5 rural areas of China from June 25, 2004, through July 15, 2008. All participants had baseline genotype data, 17 854 of whom had lipid measurements and 4657 of whom had lipoprotein particle measurements. Median follow-up of 9.2 years (interquartile range, 8.2-10.1 years) was completed January 1, 2016, through linkage to health insurance records and death and disease registries.

**Exposures:**

Five *CETP* variants, including an East Asian loss-of-function variant (rs2303790), combined in a genetic score weighted to associations with HDL cholesterol levels.

**Main Outcomes and Measures:**

Baseline levels of lipids and lipoprotein particles, cardiovascular risk factors, incidence of carotid plaque and predefined major vascular and nonvascular diseases, and a phenome-wide range of diseases.

**Results:**

Among the 151 217 individuals included in this study (58.4% women and 41.6% men), the mean (SD) age was 52.3 (10.9) years. Overall, the mean (SD) low-density lipoprotein (LDL) cholesterol level was 91 (27) mg/dL; HDL cholesterol level, 48 (12) mg/dL. *CETP* variants were strongly associated with higher concentrations of HDL cholesterol (eg, 6.1 [SE, 0.4] mg/dL per rs2303790-G allele; *P* = 9.4 × 10^−47^) but were not associated with lower LDL cholesterol levels. Within HDL particles, cholesterol esters were increased and triglycerides reduced, whereas within very low-density lipoprotein particles, cholesterol esters were reduced and triglycerides increased. When scaled to 10-mg/dL higher levels of HDL cholesterol, the *CETP* genetic score was not associated with occlusive CVD (18 550 events; odds ratio [OR], 0.98; 95% CI, 0.91-1.06), major coronary events (5767 events; OR, 1.08; 95% CI, 0.95-1.22), myocardial infarction (3118 events; OR, 1.14; 95% CI, 0.97-1.35), ischemic stroke (13 759 events; OR, 0.94; 95% CI, 0.86-1.02), intracerebral hemorrhage (6532 events; OR, 0.94; 95% CI, 0.83-1.06), or other vascular diseases or carotid plaque. Similarly, rs2303790 was not associated with any vascular diseases or plaque. No associations with nonvascular diseases were found other than an increased risk for eye diseases with rs2303790 (4090 events; OR, 1.43; 95% CI, 1.13-1.80; *P* = .003).

**Conclusions and Relevance:**

*CETP* variants were associated with altered HDL metabolism but did not lower LDL cholesterol levels and had no significant association with risk for CVD. These results suggest that in the absence of reduced LDL cholesterol levels, increasing HDL cholesterol levels by inhibition of CETP may not confer significant benefits for CVD.

## Key Points

**Question:**

What is the association of genetic variants in the *CETP* gene that lower cholesteryl ester transfer protein activity with risk for cardiovascular and other diseases?

**Findings:**

In this biobank study of 151 217 Chinese adults, *CETP* gene variants were associated with higher levels of high-density lipoprotein cholesterol but not with lower levels of low-density lipoprotein cholesterol and were not associated with risk for cardiovascular disease.

**Meaning:**

Increasing levels of high-density lipoprotein cholesterol by cholesteryl ester transfer protein inhibition in the absence of lower levels of low-density lipoprotein cholesterol may not confer significant benefits for cardiovascular disease.

## Teaser

This biobank study uses genetic variants of *CETP* to assess potential risks and benefits of lifelong lower cholesteryl ester transfer protein activity on cardiovascular diseases and other outcomes in a sample of Chinese adults.

## Introduction

Observational epidemiologic studies have reported that low plasma concentrations of high-density lipoprotein (HDL) cholesterol are an independent risk factor for occlusive cardiovascular disease (CVD), including coronary heart disease (CHD) and ischemic stroke.^1,2^ Given these associations, therapeutic strategies to reduce CVD risk by increasing HDL cholesterol concentrations have attracted considerable interest. One such approach is through pharmacologic inhibition of cholesterol ester transfer protein (CETP), which transfers esterified cholesterol from HDL to apolipoprotein B–containing lipoproteins, including very low-density lipoprotein (VLDL), in exchange for triglycerides.^3^ The first CETP inhibitor assessed in phase 3 trials, torcetrapib, was associated with increased CVD risk, probably owing to off-target effects.^4,5^ Subsequent trials of dalcetrapib (which had only modest effects on HDL cholesterol) or evacetrapib (which increased HDL cholesterol levels substantially and lowered LDL cholesterol levels) were stopped early for futility after 2 to 3 years of treatment in high-risk individuals.^6,7^ A trial of the potent CETP inhibitor anacetrapib (which doubled HDL cholesterol levels and lowered non-HDL cholesterol levels by about one-fifth) that involved approximately  30 000 high-risk individuals treated for 4 years recently reported a benefit for risk of major coronary events consistent with the effects of lowering non-HDL cholesterol levels.^8^

Genetic variants can be used to assess causal associations with a mendelian randomization approach that resembles a randomized trial because genetic variants are randomly allocated at conception and should not be subject to confounding or reverse causation bias.^9^ As such, genetic studies can be used to estimate the effects of alterations of the expression or activity of a drug target, such as CETP.^10^ Common *CETP* gene (HGNC 1869) variants associated with lower CETP mass and activity have been associated with lower risks for CHD and ischemic stroke and a higher risk for intracerebral hemorrhage.^11,12,13,14,15,16^ Previous studies were conducted mainly in populations of European origin, among whom the mean LDL cholesterol level is high compared with the Chinese population, and common *CETP* variants tend to be associated not only with higher HDL cholesterol concentrations but also with lower LDL cholesterol concentrations, as is the case for several CETP inhibitors.^17,18^ A loss-of-function variant in *CETP* (rs2303790; c.1376A>G; p.D459G) that results in lower plasma CETP levels and activity has been identified in Japanese individuals with elevated HDL cholesterol concentrations.^19,20,21^ Some studies of rs2303790 and other *CETP* loss-of-function variants suggest an association with lower CHD risk that may be mediated by lower LDL cholesterol levels, but findings are inconsistent.^22,23,24,25^

To assess the potential benefits and risks of lifelong lower CETP activity, we examined the association of *CETP* variants (rs2303790 and a genetic score consisting of this and 4 other common *CETP* variants) with lipid and lipoprotein metabolism, CVD risk factors, and a range of vascular and nonvascular diseases in as many as 151 217 adults from the China Kadoorie Biobank (CKB) study.

## Methods

### Study Population, Baseline Survey, and Resurvey

The design and methods of the CKB study have been reported in detail elsewhere.^26,27^ Overall, 512 891 adults aged 30 to 79 years were enrolled from June 25, 2004, through July 15, 2008, from 5 rural and 5 urban areas in China. CKB participants were confirmed to be of Chinese ancestry based on findings of principal component analysis of genotyping data, where available. The baseline survey included a detailed questionnaire and physical measurements (including anthropometry and blood pressure). A nonfasting blood sample was collected for on-site testing (including plasma glucose level using the SureStep Plus meter [LifeScan]) and then separated into plasma and buffy-coat fractions for long-term storage. Study procedures and staff training were standardized across regions. Periodic resurveys were conducted for approximately  5% of surviving participants. The second resurvey from August 4, 2013, through September 18, 2014, included measurements of carotid intima media thickness and plaque using a diagnostic ultrasound system (GM-72P00A; Panasonic Healthcare Co, Ltd). Ethical approval for the study was obtained from the University of Oxford, Oxford, England, the Chinese Centre for Disease Control and Prevention, and the local Centres for Disease Control and Prevention in the 10 study areas. All participants provided written informed consent.

### Long-term Follow-up

Vital status and incidence of disease events were recorded using electronic linkage of each participant’s unique national identification number with established registries for morbidity (stroke, CHD, cancer, and diabetes) and mortality in each locality and a nationwide health insurance system. Registry data included scanned copies of official death certificates and reports for hospitalization of specific diseases. Health insurance reports included detailed information (eg, disease description, *International Statistical Classification of Diseases and Related Health Problems, 10th Revision* [*ICD-10*] code, and procedure or examination codes) about each hospital admission. Events related to major chronic diseases (stroke, CHD, diabetes, chronic obstructive pulmonary disease [COPD], and cancer) were carefully reviewed and standardized. By January 1, 2016, after a median follow-up of 9.2 years (interquartile range, 8.2-10.1 years), 37 289 deaths were recorded among the 512 891 CKB participants, and 4875 (<1%) were lost to follow-up.

### Genotyping and Lipid and Lipoprotein Measurements

Five *CETP* gene variants (rs3764261, rs1800775, rs708272, rs9939224, and rs2303790; eTable 1 in the Supplement) were selected on the basis of previously reported associations with HDL cholesterol and CETP activity.^11,19,28^ Genotyping was conducted in 151 217 individuals by using a 384–single-nucleotide polymorphism (SNP) array (GoldenGate; Illumina) or a custom-designed 800K-SNP array (Axiom; Affymetrix) (call rates were >99.97% for all variants). Genotyping consisted of a population-based sample of 134 790 participants included in analyses of all disease outcomes, an additional 13 000 participants with an incident CVD event and control participants included in analyses of specified CVD outcomes, and an additional 3427 participants with an incident COPD event included in analyses of COPD. A subset of the genotyped population (17 854 selected for CVD case-control studies) had measurements of plasma concentrations of total cholesterol, LDL cholesterol, HDL cholesterol, triglycerides, lipoprotein(a), apolipoprotein B, and apolipoprotein A1 using a clinical chemistry analyzer (AU680; Beckman-Coulter). Among these individuals, 4657 also had plasma measurements of metabolomics using proton nuclear magnetic resonance spectroscopy providing data on 225 metabolic measures, including detailed lipid and lipoprotein particle profiles.^29^ Further details of assays and participants included are shown in eFigure 1 and eMethods 1 in the Supplement.

### Main Outcome Measures

Prespecified vascular outcomes included major coronary events (myocardial infarction, coronary revascularization, or death from CHD), stroke, occlusive CVD (major coronary events or ischemic stroke), major vascular events (major coronary events, stroke, or vascular-associated death), and their components (see eMethods 2 in the Supplement for *ICD-10* codes). Common controls for vascular outcomes excluded individuals reporting a history of CHD, stroke, or transient ischemic attack at baseline or any major vascular event during follow-up. Other outcomes included diabetes, COPD, chronic kidney disease, liver disease, cancer, eye disease, and nonvascular death; controls for these outcomes excluded individuals reporting a history of that disease at baseline when appropriate. Incident events in the range of *ICD-10* codes A00 to N99 were grouped into 41 distinct categories for a phenome-wide analysis using a previously described approach.^30^ For these 41 *ICD-10* categorized outcomes, no exclusions for prevalent diseases were made from controls. For all outcomes, no exclusions for prevalent diseases were made from cases (ie, not all cases were new onset), and hospital episodes were restricted to those identified from inpatient records.

### Statistical Analyses

Measurements of lipid and lipoprotein levels were stratified by area and standardized by rank inverse normal transformation after adjustment for sex and age. Continuous traits were assessed by linear regression, and disease outcomes were assessed by logistic regression with stratification by area and adjustment for sex and age. Individuals with missing genotype data were excluded from analyses of the relevant variant or the genetic score. An additive (per allele) model was used for individual variants. A multivariable model including 5 *CETP* variants was used to obtain independent per-allele associations with rank inverse normal-transformed HDL cholesterol levels, with mutual adjustment to account for linkage disequilibrium (ie, correlation) between variants (eTable 2 in the Supplement). Per-allele associations from the multivariable model (eTable 3 in the Supplement) were used to construct a weighted genetic score.^31^ Among participants with lipid-level measurements, unbiased internal weights were derived by 100-fold cross-validation. Among participants without lipid-level measurements, weights were derived directly from the multivariable model. Given the variance in HDL cholesterol levels explained by the genetic score (eTable 3 in the Supplement), the study had more than 80% power at *P* < .05 to detect a 20% lower risk for major coronary events or a 10% lower risk for major vascular events, for a 1-SD higher HDL cholesterol level. Associations of rs2303790 and the *CETP* genetic score with outcomes were scaled to correspond to 10-mg/dL higher HDL cholesterol levels (to convert to millimoles per liter, multiply by 0.0259). Subgroup analyses were performed by urban or rural area, age group, sex, smoking, and alcohol consumption. *P* values are presented as unadjusted for multiple testing, unless otherwise indicated. For assessment of significance, α = .05, a Bonferroni-corrected threshold was used that divided 0.05 by the number of outcomes examined (8 vascular, 7 nonvascular, or 41 phenome wide) or by the number of principal components accounting for 95% of variation in the proton nuclear magnetic resonance metabolomics data set (18). All analyses used SAS software (version 9.3; SAS Institute, Inc).

## Results

Among the 151 217 individuals included in this study, the mean (SD) age was 52.3 (10.9) years. A total of 58.4% were women and 41.6% were men; 42.0% were from urban areas (Table). Compared with controls, individuals reporting a major vascular event during follow-up were older, less likely to be female, and more likely to reside in urban areas (eTable 4 in the Supplement). In a subset selected for CVD case-control studies with no self-reported history of CVD or treatment to lower lipid levels at baseline, the mean (SD) baseline plasma HDL cholesterol concentration was 48 (12) mg/dL; LDL cholesterol concentration, 91 (27) mg/dL; and total cholesterol concentration, 180 (38) mg/dL. Median triglyceride concentration was 139.8 mg/dL (interquartile range, 95.6-211.5 mg/dL; to convert to millimoles per liter, multiply by 0.0113).

**Table.** Selected Baseline Characteristics of the Study Population

Characteristic	Data (N = 151 217)
Age, mean (SD), y	52.3 (10.9)
Female, No. (%)	88 361 (58.4)
Urban area, No. (%)	63 447 (42.0)
Educational attainment >6 y, No. (%)	30 018 (19.9)
Income >20 000 yuan/y, No. (%)^a^	60 936 (40.3)
Disease history, No. (%)	
Hypertension	18 731 (12.4)
CHD	4534 (3.0)
Stroke or transient ischemic attack	2358 (1.6)
Diabetes	5145 (3.4)
Medication use, No. (%)	
Antihypertensives	7616 (5.0)
Statins	332 (0.2)
Regular smoking, No. (%)	40 634 (26.9)
Regular alcohol consumption, No. (%)	22 742 (15.0)
Physical activity, mean (SD), MET-h/d	20.7 (13.9)
Systolic blood pressure (SD), mm Hg	132.5 (22.1)
Standing height, mean (SD), cm	158.6 (82.8)
Body mass index, mean (SD)^b^	23.6 (3.4)
Waist circumference, mean (SD), cm	80.2 (9.9)
Random plasma glucose level, mean (SD), mg/dL^c^	109.9 (43.2)
Lipid and lipoprotein levels, mean (SD)^d^	
HDL cholesterol	47.7 (11.5)
LDL cholesterol	91.4 (27.4)
Total cholesterol	180.0 (38.3)
Lipoprotein(a)	1.04 (1.31)
Apolipoprotein A1	134.1 (22.3)
Apolipoprotein B	83.8 (21.2)
Triglycerides, median (IQR)	139.8 (95.6-211.5)

Abbreviations: CHD, coronary heart disease; CVD, cardiovascular disease; HDL, high-density lipoprotein; IQR, interquartile range; LDL, low-density lipoprotein; MET, metabolic task equivalent.

SI conversion factors: To convert cholesterol to millimoles per liter, multiply by 0.0259; glucose to millimoles per liter, multiply by 0.0555; lipoprotein(a) to micromoles per liter, multiply by 0.0357; lipoproteins A1 and B to grams per liter, multiply by 0.01; and triglycerides to millimoles per liter, multiply by 0.0113.

^a^ One yuan equals US $0.15.

^b^ Calculated as weight in kilograms divided by height in meters squared.

^c^ Measured in 148 693 individuals.

^d^ Measured by clinical biochemistry in a selected subset of 17 854 individuals with incident CVD and control individuals with no history of CVD at baseline and not using statin treatment. Unless otherwise indicated, data are reported as milligrams per deciliter.

The *CETP* loss-of-function variant rs2303790-G (allele frequency, 2%; eTable 1 in the Supplement) was associated with 6.1-mg/dL (SE, 0.4-mg/dL) higher HDL cholesterol levels per allele (equivalent to 0.53 of the SD; *P* = 9.4 × 10^−47^) (eTable 5 in the Supplement). The 4 common *CETP* variants were also associated with higher HDL cholesterol levels (1.4-3.6 mg/dL per allele; allele frequencies, 16%-88%). In a joint model, all 5 variants had independent associations with HDL cholesterol level (0.7-4.0 mg/dL per allele) (eTable 3 in the Supplement), and in the absence of measured CETP activity, a genetic score was weighted according to these HDL cholesterol associations.

Baseline characteristics of the study participants, including age, income, smoking, and alcohol drinking, did not vary significantly by rs2303790 genotype or the *CETP* genetic score after adjustment for sex, age, and area (eTable 6 in the Supplement), indicating that analyses of rs2303790 and the genetic score were not confounded by these factors. However, the prevalence of previously diagnosed hypertension varied across the tertiles of the genetic score (12.8% vs 11.9% for the lowest compared with highest tertile; *P* = 3.5 × 10^−5^ for trend).

**Figure 1.**
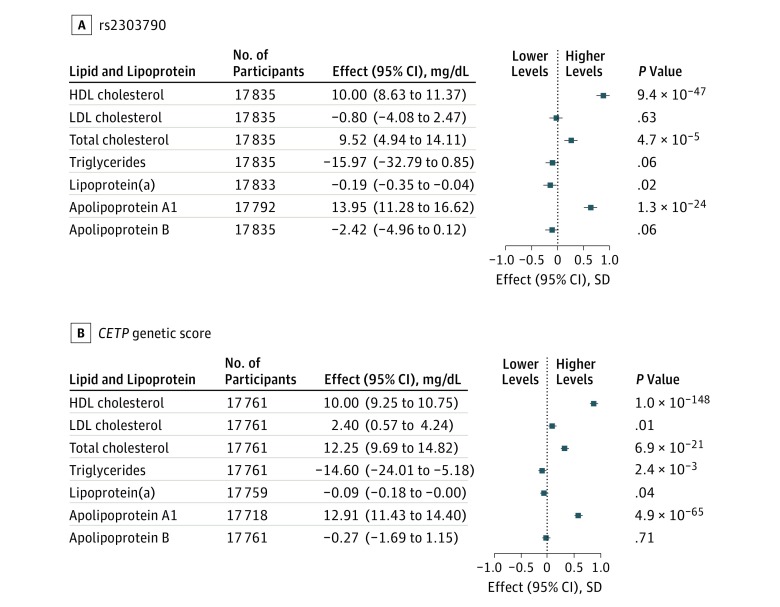
Associations of rs2303790 and a *CETP* Genetic Score With Lipids and Lipoproteins Measured by Clinical Biochemical Analysis The association of rs2303790 and a *CETP* genetic score (consisting of rs3764261, rs1800775, rs708272, rs9939224, and rs2303790) with rank inverse normal transformation–standardized traits measured by clinical biochemical analysis in a subset of 17 854 individuals was scaled to 10-mg/dL higher levels of high-density lipoprotein (HDL) cholesterol. Findings were adjusted for sex and age and stratified by study area. Further adjustment for time since the last meal or cardiovascular disease case or control status had no appreciable effect on the associations. Squares represent the associations in standard deviations of each trait. Error bars represent the corresponding 95% CIs. *P* values are not adjusted for multiple testing. To convert cholesterol to millimoles per liter, multiply by 0.0259; lipoprotein(a) to micromoles per liter, multiply by 0.0357; lipoproteins A1 and B to grams per liter, multiply by 0.01; and triglycerides to millimoles per liter, multiply by 0.0113. LDL indicates low-density lipoprotein.

The loss-of-function variant rs2303790 was not associated with LDL cholesterol or triglyceride levels but was associated with 0.19 mg/dL (95% CI, 0.04-0.35 mg/dL) lower lipoprotein(a) levels when scaled to a 10-mg/dL higher HDL cholesterol level (Figure 1). The *CETP* genetic score, similarly scaled to 10-mg/dL higher HDL cholesterol levels, was associated with 2.4-mg/dL (95% CI, 0.6- to 4.2-mg/dL) higher LDL cholesterol levels, 14.6-mg/dL (95% CI, 5.2- to 24.0-mg/dL) lower triglyceride levels, and 0.09-mg/dL (95% CI, 0.00- to 0.18-mg/dL) lower lipoprotein(a) levels. The 4 common *CETP* variants assessed individually were all associated with higher LDL cholesterol levels (0.6-1.2 mg/dL per allele) (eTable 5 in the Supplement), and all except rs9939224 were associated with lower triglyceride levels (3.1-4.9 mg/dL per allele). When assessed separately by area, the associations of rs2303790 or the genetic score with LDL cholesterol level were not related to the mean LDL cholesterol level in each area (eTable 7 in the Supplement).

**Figure 2.**
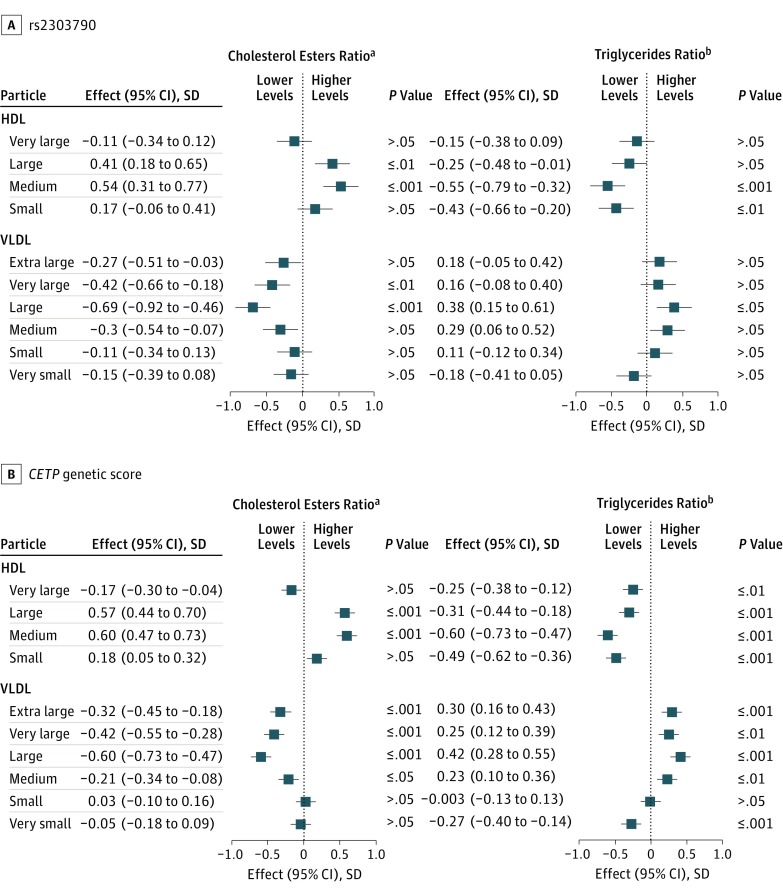
Associations of rs2303790 and a *CETP* Genetic Score With Lipoprotein Particle Composition Measured by Proton Nuclear Magnetic Resonance (NMR) Metabolomics The association of rs2303790 and a *CETP* genetic score (consisting of rs3764261, rs1800775, rs708272, rs9939224, and rs2303790) with rank inverse normal transformation–standardized traits measured by NMR metabolomics was scaled to 10-mg/dL higher levels of high-density lipoprotein (HDL) cholesterol. Findings were adjusted for sex and age and stratified by study area. NMR measurements were performed for 4657 individuals, but data for these analyses were available for 4422 to 4652 participants after exclusions for missing data for individual traits and genotypes. Squares represent the associations in standard deviations of each trait. Error bars represent the corresponding 95% CIs. *P* values were calculated after Bonferroni adjustment for 18 principal components among the 225 measured NMR traits. VLDL indicates very low-density lipoprotein. ^a^Data are presented as the ratio of cholesterol esters to total lipids in lipoprotein particle subtypes. ^b^Data are presented as the ratio of triglycerides to total lipids in lipoprotein particle subtypes.

We found similar patterns of association for rs2303790 and the *CETP* genetic score with the lipid compositions of lipoprotein particles measured by proton nuclear magnetic resonance metabolomics. Consistent with the expected associations of lower CETP activity (ie, a genetic proxy for CETP inhibition), *CETP* variants that increased HDL cholesterol levels were associated with higher levels of esterified cholesterol within large and medium HDL particles and lower levels within extra large, very large, and large VLDL particles relative to the total lipid content of these particles (Figure 2). Conversely, levels of triglycerides relative to total lipids were higher in VLDL particles and lower in HDL particles. Furthermore, HDL particle size was larger and LDL particle size smaller, and the concentration of mature (large and very large) HDL particles was higher (eFigure 2 in the Supplement). The overall concentration of cholesterol in HDL and LDL particles was higher and, in VLDL particles, was lower.

In analyses of continuous traits, the *CETP* genetic score was associated with lower systolic blood pressure of 0.74 (SE, 0.25) mm Hg per 10-mg/dL higher HDL cholesterol level (*P* = .004) (eTable 8 in the Supplement). Neither rs2303790 nor the *CETP* genetic score was associated with body mass index, waist circumference, or random plasma glucose levels, nor were they associated with carotid intima media thickness or carotid plaque.

**Figure 3.**
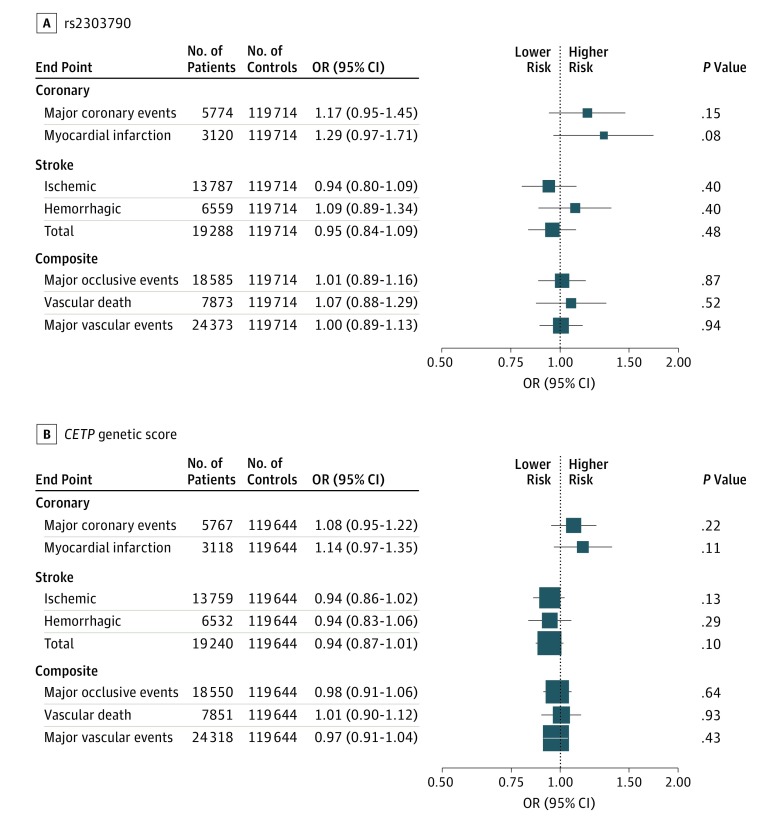
Associations of rs2303790 and a *CETP* Genetic Score With Vascular Diseases The association of rs2303790 and a *CETP* genetic score (consisting of rs3764261, rs1800775, rs708272, rs9939224, and rs2303790) with vascular diseases was scaled to 10-mg/dL higher levels of high-density lipoprotein cholesterol. Findings were adjusted for sex and age and stratified by study area. Squares represent the odds ratio (OR) with area inversely proportional to the variance of the logarithm OR. Error bars represent the corresponding 95% CIs. *P* values in the plot are not adjusted for multiple testing, but Bonferroni adjustment for 8 outcomes would result in a threshold of *P* < .0063 (.05/8).

We found no associations of rs2303790 or the *CETP* genetic score with risk for major vascular diseases (Figure 3). For major occlusive CVD events, the adjusted odds ratios (ORs) were 1.01 (95% CI, 0.89-1.16; 18 585 events) for rs2303790 and 0.98 (95% CI, 0.91-1.06; 18 550 events) for the genetic score, both scaled to 10-mg/dL higher HDL cholesterol levels. The *CETP* genetic score was not associated with the components of occlusive CVD, including major coronary events (OR, 1.08; 95% CI, 0.95-1.22; 5767 events) and ischemic stroke (OR, 0.94; 95% CI, 0.86-1.02; 13 759 events). Similarly, we found no associations of the genetic score with myocardial infarction (OR, 1.14; 95% CI, 0.97-1.35), intracerebral hemorrhage (OR, 0.94; 95% CI, 0.83-1.06), total stroke (OR, 0.94; 95% CI, 0.87-1.01), vascular death (OR, 1.01; 95% CI, 0.90-1.12), or major vascular events (OR, 0.97; 95% CI, 0.91-1.04). Estimates for rs2303790 were similar. We found no differences in the associations of the *CETP* genetic score with occlusive CVD among several subgroups (eTable 9 in the Supplement). Adjusting for systolic blood pressure had no material effect on the association of the genetic score with occlusive CVD.

**Figure 4.**
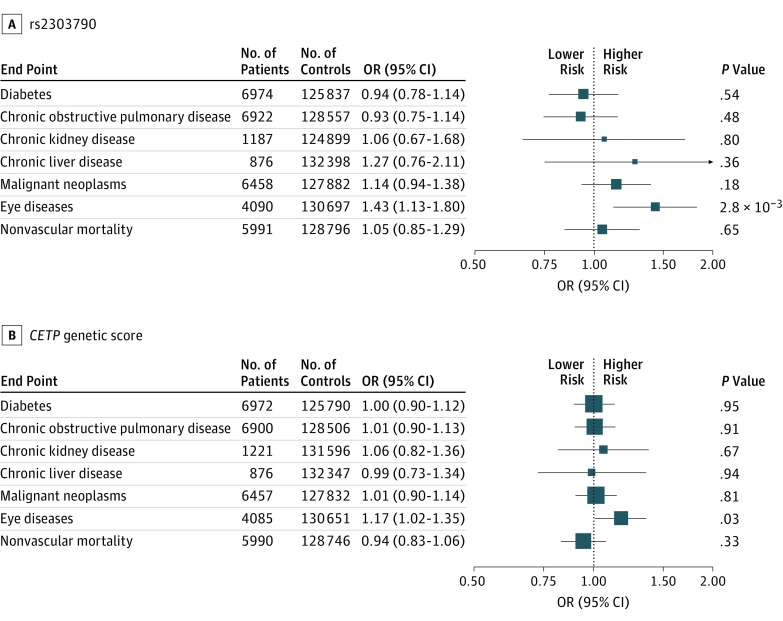
Associations of rs2303790 and a *CETP* Genetic Score With Nonvascular Diseases The association of rs2303790 and a *CETP* genetic score (consisting of rs3764261, rs1800775, rs708272, rs9939224, and rs2303790) with nonvascular diseases was scaled to 10-mg/dL higher levels of high-density lipoprotein cholesterol. Findings were adjusted for sex and age and stratified by study area. Squares represent the odds ratio (OR) with area inversely proportional to the variance of the logarithm OR. Error bars represent the corresponding 95% CIs. *P* values in the plot are not adjusted for multiple testing, but Bonferroni adjustment for 7 outcomes would result in a threshold of *P* < .0071 (.05/7).

No associations were observed for diabetes, COPD, chronic kidney disease, cancer, and nonvascular death (Figure 4). However, a higher risk for eye diseases was found with rs2303790 (OR, 1.43; 95% CI, 1.13-1.80; *P* = .003), which was significant after adjustment for multiple testing. Of 4090 eye disease events, 2980 were cataracts, and rs2303790 showed the same direction of association with cataracts (OR, 1.43; 95% CI, 1.09-1.88; *P* = .01) as with noncataract eye diseases (OR, 1.53; 95% CI, 0.99-2.35; *P* = .06). The association of the *CETP* genetic score with eye diseases was directionally consistent (OR, 1.17; 95% CI, 1.02-1.35; *P* = .03) but was not significant after correction for multiple testing. Analyses of age-related macular degeneration suggested a direction of association (OR, 1.39; 95% CI, 0.42-4.44 for the genetic score) consistent with previous reports of the association of age-related macular degeneration with *CETP* gene variants; however, rs2303790 could not be reliably assessed owing to the low allele frequency and limited number of cases (70 reported among genotyped participants).^24,32,33^ In the phenome-wide screen, we found no associations of the *CETP* genetic score with any of the 41 *ICD-10* coded disease categories, including diseases of the nervous system (OR, 1.49; 95% CI, 1.16-1.92; *P* = .002), after correction for multiple testing (Bonferroni-corrected threshold *P* = .05 for 41 disease categories, *P* = .001) (eFigure 3 in the Supplement).

## Discussion

This large genetic study of 151 217 Chinese adults found no evidence to support a beneficial association with CVD of increasing HDL cholesterol concentration through CETP inhibition. Four common *CETP* variants and an East Asian loss-of-function variant were associated with higher HDL cholesterol levels but did not lower LDL cholesterol levels, as seen in previous genetic studies performed mainly in European populations and with pharmacologic CETP inhibitors.^7,8,17^ These genetic variants influenced lipid and lipoprotein particle metabolism in a manner consistent with lower CETP activity, including reduced CETP-mediated movement of esterified cholesterol from mature HDL particles to VLDL in parallel with reduced movement of triglycerides from VLDL to HDL. However, we found no significant association of the loss-of-function variant rs2303790 or a *CETP* genetic score with the risk of occlusive CVD, major coronary events, stroke subtypes, or other major vascular diseases. When we assessed a range of predefined nonvascular diseases to identify other potential risks and benefits of CETP inhibition, rs2303790 was associated with an increased risk for eye diseases.

Common *CETP* variants have been associated with a modest lower risk for CHD, mainly in populations of European origin, including recent large studies that reported an approximately 5% lower risk with genetic variants that increased HDL cholesterol levels.^12,15,16^ These results are in contrast to the present null findings. Common *CETP* variants were also associated with an almost 2-fold increased risk for intracerebral hemorrhage in a meta-analysis involving 2800 cases of European origin,^14^ but with 6500 cases, we found no such association with intracerebral hemorrhage. Rare protein-truncating variants in populations of East Asian and European ancestry have been associated with lower CHD risk, and 2 studies in East Asians involving a total of 5082 cases reported an approximately 17% lower risk for CHD with rs2303790.^23,24,25^ However, when published data for rs2303790 were meta-analyzed with results from the present study, no significant association was evident (for 10 856 coronary events, OR, 0.97; 95% CI, 0.88-1.07) (eTable 10 in the Supplement) nor was there any association with the intermediate CVD traits carotid thickness and plaque. Of note, coronary events in the present study population showed the expected associations with variants at 9p21 (eTable 11 in the Supplement).

The association of *CETP* variants with CVD risk in previous studies^16,25^ may have been influenced, partly or wholly, by lower LDL cholesterol level or other lipid-related factors rather than higher HDL cholesterol level. The association of common *CETP* variants with LDL cholesterol levels in the present study were consistent with other studies in East Asians^28^ but directionally different from previous studies in Europeans^17^ (eTable 12 in the Supplement). Differences in LDL cholesterol level measurement methods may have contributed to such discrepancies because most previous studies contributing to the large European consortia^17^ estimated LDL cholesterol level using the Friedewald formula in contrast to the present study, which measured LDL cholesterol level directly. A study in Japanese adults also using the Friedewald formula^23^ found that rs2303790 was associated with 0.2-SD lower LDL cholesterol level, an association not seen in the present study. If the composition of VLDL particles is altered, as with genetic or pharmacologic CETP inhibition, then this alteration may affect the comparability of LDL cholesterol levels measured directly or estimated using the Friedewald formula.^34^

Although inverse associations between HDL cholesterol concentration and occlusive CVD have been widely reported in large prospective studies,^1,2^ including the CKB,^35^ the causal relevance of such associations has not been established.^12,36,37^ In a prospective study, HDL efflux capacity was inversely associated with atherosclerotic CVD risk in a population in which HDL cholesterol concentration had no significant association.^38^ Another recent study reported that a functional variant in the scavenger receptor B1 (*SRB1*) gene, which blocks uptake of HDL-associated cholesterol into the liver, was associated with higher HDL cholesterol level and increased CHD risk.^39^ Any associations of elevated HDL cholesterol level with vascular disease may vary depending on the mechanisms involved and may not be beneficial if aspects of reverse cholesterol transport, such as cholesterol efflux, or other important functions of HDL are impeded.

With linkage to electronic health records in a large prospective study, we were able to assess the associations of *CETP* genetic variants with a range of diseases, which could identify other potential beneficial or adverse associations with lifelong lower CETP activity. The risk for eye disease was elevated with rs2303790, with weaker but directionally consistent findings for the *CETP* genetic score. In a recent genome-wide study of age-related macular degeneration in East Asians,^24^ the strongest association signal was observed for rs2303790 (OR, 1.70; *P* = 5.6 × 10^−22^). Other studies of age-related macular degeneration in East Asians and Europeans^32,33,40^ have identified associations at *CETP* and other loci associated with HDL cholesterol levels, suggesting that higher HDL cholesterol level or other changes may be associated with an increased risk for age-related macular degeneration. The present study had only a limited number of reported age-related macular degeneration cases, but the direction of association with *CETP* variants was consistent with previous reports. These results suggest that CETP inhibition may have a potential adverse association with eye diseases.

### Strengths and Limitations

Genetic studies are a useful tool in drug development, specifically by prioritizing targets, assessing safety, and identifying opportunities for alternative indications.^10^ Although the present study did not measure CETP levels or activity, the genetic associations with lipid and lipoprotein metabolism were consistent with lower CETP activity and suggest that increasing HDL cholesterol levels through this pathway may not be associated with reduced CVD risk. Pharmacologic CETP inhibitors, however, have more potent effects to raise HDL cholesterol levels than genetic variants, as well as other potentially favorable lipid modifications, including lowering LDL cholesterol levels.^7,8,18^ In contrast, in the present study, LDL cholesterol level was modestly increased in association with the *CETP* genetic score. Genetic studies are also limited to assessing on-target drug effects and are not able to identify off-target toxic effects, such as the increased blood pressure seen with torcetrapib (blood pressure was also slightly increased with other CETP inhibitors).^4,5,6,7,8^ Systolic blood pressure was, in contrast, modestly lower with *CETP* variants in the present study. The present study provides important new evidence about the relevance of increasing HDL cholesterol levels through lower CETP activity and complements findings from the Randomized Evaluation of the Effects of Anacetrapib Through Lipid Modification (REVEAL) trial,^8^ in which the approximately 10% lower risk for major coronary events was consistent with the observed reduction in non-HDL cholesterol levels, suggesting that the benefits were not driven by increasing HDL cholesterol levels.

## Conclusions

Genetic variants in the *CETP* gene that were associated with altered HDL metabolism but not lower LDL cholesterol levels had no association with CVD risk in 151 217 Chinese adults. These results suggest that in the absence of significantly reduced LDL cholesterol, increasing HDL cholesterol levels by CETP inhibition may not be associated with reduced risk for CVD.

## Notes

Supplement. **eTable 1.** Allele Frequencies of 5 *CETP* Genetic Variants **eTable 2.** Pairwise Linkage Disequilibrium Among 5 *CETP* Genetic Variants **eTable 3.** Derivation of a *CETP* Genetic Score Weighted by Independent Effects on HDL Cholesterol **eTable 4.** Selected Baseline Characteristics Among Major Vascular Disease Cases and Common Vascular Disease Controls **eTable 5.** Associations of *CETP* Genetic Variants With Lipids and Lipoproteins Measured by Clinical Biochemistry **eTable 6.** Baseline Characteristics of the Study Population by rs2303790 Genotype and a *CETP* Genetic Score **eTable 7.** Association of rs2303790 and a *CETP* Genetic Score, Both Scaled to 10-mg/dL Higher HDL Cholesterol, With LDL Cholesterol, According to Mean LDL Cholesterol in 10 Study Areas **eTable 8.** Associations of rs2303790 and a *CETP* Genetic Score, Both Scaled to 10-mg/dL Higher HDL Cholesterol, With Continuous Traits Measured at Baseline and Carotid Intima Media Thickness and Plaque Measured at the Second Survey **eTable 9.** Associations of a *CETP* Genetic Score, Scaled to 10-mg/dL Higher HDL Cholesterol, With Occlusive CVD by Subgroups **eTable 10.** Meta-analysis of rs2303790 With Coronary Heart Disease in the CKB and 2 Published Studies **eTable 11.** Association of rs1333049 at the 9p21 Locus With Coronary Events in CKB **eTable 12.** Comparison of the Associations of *CETP* Genetic Variants With Lipids in the CKB and the Global Lipids Genetics Consortium **eFigure 1.** Study Participant Flowchart **eFigure 2.** Associations of rs2303790 and a *CETP* Genetic Score With Lipoprotein Particle Size, Concentration, and Cholesterol Concentration **eFigure 3.** Associations of a *CETP* Genetic Score With a Phenome-Wide Screen of 41 Disease Categories **eMethods 1.** Details of Genotyping and Lipid and Lipoprotein Measurements **eMethods 2.** Disease Outcomes and *ICD-10* Codes

## References

[1] Lewington S, Whitlock G, Clarke R, ; Prospective Studies Collaboration Blood cholesterol and vascular mortality by age, sex, and blood pressure: a meta-analysis of individual data from 61 prospective studies with 55,000 vascular deaths. Lancet. 2007;370(9602):1829-1839. 18061058 10.1016/S0140-6736(07)61778-4

[2] Di Angelantonio E, Sarwar N, Perry P, ; Emerging Risk Factors Collaboration Major lipids, apolipoproteins, and risk of vascular disease. JAMA. 2009;302(18):1993-2000. 19903920 10.1001/jama.2009.1619 PMC3284229

[3] Tall AR Plasma cholesteryl ester transfer protein. J Lipid Res. 1993;34(8):1255-1274. 8409761

[4] Barter PJ, Caulfield M, Eriksson M, ; ILLUMINATE Investigators Effects of torcetrapib in patients at high risk for coronary events. N Engl J Med. 2007;357(21):2109-2122. 17984165 10.1056/NEJMoa0706628

[5] Johns DG, Duffy J, Fisher T, Hubbard BK, Forrest MJ On- and off-target pharmacology of torcetrapib: current understanding and implications for the structure activity relationships (SAR), discovery and development of cholesteryl ester-transfer protein (CETP) inhibitors. Drugs. 2012;72(4):491-507. 22356288 10.2165/11599310-000000000-00000

[6] Schwartz GG, Olsson AG, Abt M, ; dal-OUTCOMES Investigators Effects of dalcetrapib in patients with a recent acute coronary syndrome. N Engl J Med. 2012;367(22):2089-2099. 23126252 10.1056/NEJMoa1206797

[7] Lincoff AM, Nicholls SJ, Riesmeyer JS, ; ACCELERATE Investigators Evacetrapib and cardiovascular outcomes in high-risk vascular disease. N Engl J Med. 2017;376(20):1933-1942. 28514624 10.1056/NEJMoa1609581

[8] HPS3/TIMI55-REVEAL Collaborative Group Effects of anacetrapib in patients with atherosclerotic vascular disease. N Engl J Med. 2017;377(13):1217-1227. 28847206 10.1056/NEJMoa1706444

[9] Evans DM, Davey Smith G Mendelian randomization: new applications in the coming age of hypothesis-free causality. Annu Rev Genomics Hum Genet. 2015;16:327-350. 25939054 10.1146/annurev-genom-090314-050016

[10] Plenge RM, Scolnick EM, Altshuler D Validating therapeutic targets through human genetics. Nat Rev Drug Discov. 2013;12(8):581-594. 23868113 10.1038/nrd4051

[11] Thompson A, Di Angelantonio E, Sarwar N, . Association of cholesteryl ester transfer protein genotypes with CETP mass and activity, lipid levels, and coronary risk. JAMA. 2008;299(23):2777-2788. 18560005 10.1001/jama.299.23.2777

[12] Voight BF, Peloso GM, Orho-Melander M, . Plasma HDL cholesterol and risk of myocardial infarction: a mendelian randomisation study. Lancet. 2012;380(9841):572-580. 22607825 10.1016/S0140-6736(12)60312-2 PMC3419820

[13] Johannsen TH, Frikke-Schmidt R, Schou J, Nordestgaard BG, Tybjærg-Hansen A Genetic inhibition of *CETP*, ischemic vascular disease and mortality, and possible adverse effects. J Am Coll Cardiol. 2012;60(20):2041-2048. 23083790 10.1016/j.jacc.2012.07.045

[14] Anderson CD, Falcone GJ, Phuah CL, ; Global Lipids Genetics Consortium and International Stroke Genetics Consortium. Genetic variants in *CETP* increase risk of intracerebral hemorrhage. Ann Neurol. 2016;80(5):730-740. 27717122 10.1002/ana.24780 PMC5115931

[15] Webb TR, Erdmann J, Stirrups KE, ; Wellcome Trust Case Control Consortium; MORGAM Investigators; Myocardial Infarction Genetics and CARDIoGRAM Exome Consortia Investigators Systematic evaluation of pleiotropy identifies 6 further loci associated with coronary artery disease. J Am Coll Cardiol. 2017;69(7):823-836. 28209224 10.1016/j.jacc.2016.11.056 PMC5314135

[16] Ference BA, Kastelein JJP, Ginsberg HN, . Association of genetic variants related to CETP inhibitors and statins with lipoprotein levels and cardiovascular risk. JAMA. 2017;318(10):947-956. 28846118 10.1001/jama.2017.11467 PMC5710502

[17] Willer CJ, Schmidt EM, Sengupta S, ; Global Lipids Genetics Consortium Discovery and refinement of loci associated with lipid levels. Nat Genet. 2013;45(11):1274-1283. 24097068 10.1038/ng.2797 PMC3838666

[18] Rader DJ, deGoma EM Future of cholesteryl ester transfer protein inhibitors. Annu Rev Med. 2014;65:385-403. 24422575 10.1146/annurev-med-050311-163305

[19] Takahashi K, Jiang XC, Sakai N, A missense mutation in the cholesteryl ester transfer protein gene with possible dominant effects on plasma high density lipoproteins. J Clin Invest. 1993;92(4):2060-2064. 8408659 10.1172/JCI116802 PMC288375

[20] Inazu A, Jiang XC, Haraki T, . Genetic cholesteryl ester transfer protein deficiency caused by two prevalent mutations as a major determinant of increased levels of high density lipoprotein cholesterol. J Clin Invest. 1994;94(5):1872-1882. 7962532 10.1172/JCI117537 PMC305391

[21] Nagano M, Yamashita S, Hirano K, . Molecular mechanisms of cholesteryl ester transfer protein deficiency in Japanese. J Atheroscler Thromb. 2004;11(3):110-121. 15256762 10.5551/jat.11.110

[22] Zhong S, Sharp DS, Grove JS, . Increased coronary heart disease in Japanese-American men with mutation in the cholesteryl ester transfer protein gene despite increased HDL levels. J Clin Invest. 1996;97(12):2917-2923. 8675707 10.1172/JCI118751 PMC507389

[23] Takeuchi F, Isono M, Katsuya T, . Association of genetic variants influencing lipid levels with coronary artery disease in Japanese individuals. PLoS One. 2012;7(9):e46385. 23050023 10.1371/journal.pone.0046385 PMC3458872

[24] Cheng CY, Yamashiro K, Chen LJ, . New loci and coding variants confer risk for age-related macular degeneration in East Asians. Nat Commun. 2015;6:6063. 25629512 10.1038/ncomms7063 PMC4317498

[25] Nomura A, Won HH, Khera AV, . Protein-truncating variants at the cholesteryl ester transfer protein gene and risk for coronary heart disease. Circ Res. 2017;121(1):81-88. 28506971 10.1161/CIRCRESAHA.117.311145 PMC5523940

[26] Chen Z, Lee L, Chen J, . Cohort profile: the Kadoorie Study of Chronic Disease in China (KSCDC). Int J Epidemiol. 2005;34(6):1243-1249. 16131516 10.1093/ije/dyi174

[27] Chen Z, Chen J, Collins R, ; China Kadoorie Biobank (CKB) Collaborative Group China Kadoorie Biobank of 0.5 million people: survey methods, baseline characteristics and long-term follow-up. Int J Epidemiol. 2011;40(6):1652-1666. 22158673 10.1093/ije/dyr120 PMC3235021

[28] Teslovich TM, Musunuru K, Smith AV, . Biological, clinical and population relevance of 95 loci for blood lipids. Nature. 2010;466(7307):707-713. 20686565 10.1038/nature09270 PMC3039276

[29] Soininen P, Kangas AJ, Würtz P, . High-throughput serum NMR metabonomics for cost-effective holistic studies on systemic metabolism. Analyst. 2009;134(9):1781-1785. 19684899 10.1039/b910205a

[30] Millwood IY, Bennett DA, Walters RG, ; China Kadoorie Biobank Collaborative Group A phenome-wide association study of a lipoprotein-associated phospholipase A2 loss-of-function variant in 90 000 Chinese adults. Int J Epidemiol. 2016;45(5):1588-1599. 27301456 10.1093/ije/dyw087 PMC5100610

[31] Burgess S, Thompson SG Use of allele scores as instrumental variables for Mendelian randomization. Int J Epidemiol. 2013;42(4):1134-1144. 24062299 10.1093/ije/dyt093 PMC3780999

[32] Momozawa Y, Akiyama M, Kamatani Y, . Low-frequency coding variants in CETP and CFB are associated with susceptibility of exudative age-related macular degeneration in the Japanese population. Hum Mol Genet. 2016;25(22):5027-5034. 28173125 10.1093/hmg/ddw335

[33] Burgess S, Davey Smith G Mendelian randomization implicates high-density lipoprotein cholesterol–associated mechanisms in etiology of age-related macular degeneration. Ophthalmology. 2017;124(8):1165-1174. 28456421 10.1016/j.ophtha.2017.03.042 PMC5526457

[34] Davidson M, Liu SX, Barter P, . Measurement of LDL-C after treatment with the CETP inhibitor anacetrapib. J Lipid Res. 2013;54(2):467-472. 23172660 10.1194/jlr.M032615 PMC3588873

[35] Holmes MV, Millwood IY, Kartsonaki C, Serum NMR metabolomics identifies similar associations of lipoproteins and lipids with risk of myocardial infarction and ischemic stroke but not with hemorrhagic stroke. Circulation. 2016;134:A14009.

[36] Holmes MV, Asselbergs FW, Palmer TM, ; UCLEB Consortium Mendelian randomization of blood lipids for coronary heart disease. Eur Heart J. 2015;36(9):539-550. 24474739 10.1093/eurheartj/eht571 PMC4344957

[37] White J, Swerdlow DI, Preiss D, . Association of lipid fractions with risks for coronary artery disease and diabetes. JAMA Cardiol. 2016;1(6):692-699. 27487401 10.1001/jamacardio.2016.1884 PMC5642865

[38] Rohatgi A, Khera A, Berry JD, . HDL cholesterol efflux capacity and incident cardiovascular events. N Engl J Med. 2014;371(25):2383-2393. 25404125 10.1056/NEJMoa1409065 PMC4308988

[39] Zanoni P, Khetarpal SA, Larach DB, ; CHD Exome+ Consortium; CARDIoGRAM Exome Consortium; Global Lipids Genetics Consortium Rare variant in scavenger receptor BI raises HDL cholesterol and increases risk of coronary heart disease. Science. 2016;351(6278):1166-1171. 26965621 10.1126/science.aad3517 PMC4889017

[40] Chen W, Stambolian D, Edwards AO, ; Complications of Age-Related Macular Degeneration Prevention Trial Research Group Genetic variants near *TIMP3* and high-density lipoprotein–associated loci influence susceptibility to age-related macular degeneration. Proc Natl Acad Sci U S A. 2010;107(16):7401-7406. 20385819 10.1073/pnas.0912702107 PMC2867722

