# Supplementary material for: Association of CETP Gene Variants With Risk for Vascular and Nonvascular Diseases Among Chinese Adults
Source: JAMA Cardiol. 2017 Nov 15;3(1):34–43. doi: 10.1001/jamacardio.2017.4177 (PMC5833522; doi:10.1001/jamacardio.2017.4177)
Supplement: Supplement. — eTable 1. Allele Frequencies of 5 CETP Genetic Variants eTable 2. Pairwise Linkage Disequilibrium Among 5 CETP Genetic Variants eTable 3. Derivation of a CETP Genetic Score Weighted by Independent Effects on HDL Cholesterol eTable 4. Selected Baseline Characteristics Among Major Vascular Disease Cases and Common Vascular Disease Controls eTable 5. Associations of CETP Genetic Variants With Lipids and Lipoproteins Measured by Clinical Biochemistry eTable 6. Baseline Characteristics of the Study Population by rs2303790 Genotype and a CETP Genetic Score eTable 7. Association of rs2303790 and a CETP Genetic Score, Both Scaled to 10-mg/dL Higher HDL Cholesterol, With LDL Cholesterol, According to Mean LDL Cholesterol in 10 Study Areas eTable 8. Associations of rs2303790 and a CETP Genetic Score, Both Scaled to 10-mg/dL Higher HDL Cholesterol, With Continuous Traits Measured at Baseline and Carotid Intima Media Thickness and Plaque Measured at the Second Survey eTable 9. Associations of a CETP Genetic Score, Scaled to 10-mg/dL Higher HDL Cholesterol, With Occlusive CVD by Subgroups eTable 10. Meta-analysis of rs2303790 With Coronary Heart Disease in the CKB and 2 Published Studies eTable 11. Association of rs1333049 at the 9p21 Locus With Coronary Events in CKB eTable 12. Comparison of the Associations of CETP Genetic Variants With Lipids in the CKB and the Global Lipids Genetics Consortium eFigure 1. Study Participant Flowchart eFigure 2. Associations of rs2303790 and a CETP Genetic Score With Lipoprotein Particle Size, Concentration, and Cholesterol Concentration eFigure 3. Associations of a CETP Genetic Score With a Phenome-Wide Screen of 41 Disease Categories eMethods 1. Details of Genotyping and Lipid and Lipoprotein Measurements eMethods 2. Disease Outcomes and ICD-10 Codes [file jamacardiol-3-34-s001.pdf]

Supplementary Online Content

Millwood IY, Bennett DA, Holmes MV, et al; China Kadoorie Biobank Collaborative Group. Association of *CETP* gene variants with risk for vascular and nonvascular diseases among Chinese adults. *JAMA Cardiol*. Published online November 15, 2017. doi:10.1001/jamacardio.2017.4177

eTable 1. Allele Frequencies of 5 *CETP* Genetic Variants
eTable 2. Pairwise Linkage Disequilibrium Among 5 *CETP* Genetic Variants
eTable 3. Derivation of a *CETP* Genetic Score Weighted by Independent Effects on HDL Cholesterol
eTable 4. Selected Baseline Characteristics Among Major Vascular Disease Cases and Common Vascular Disease Controls
eTable 5. Associations of *CETP* Genetic Variants With Lipids and Lipoproteins Measured by Clinical Biochemistry
eTable 6. Baseline Characteristics of the Study Population by rs2303790 Genotype and a *CETP* Genetic Score
eTable 7. Association of rs2303790 and a *CETP* Genetic Score, Both Scaled to 10-mg/dL Higher HDL Cholesterol, With LDL Cholesterol, According to Mean LDL Cholesterol in 10 Study Areas
eTable 8. Associations of rs2303790 and a *CETP* Genetic Score, Both Scaled to 10-mg/dL Higher HDL Cholesterol, With Continuous Traits Measured at Baseline and Carotid Intima Media Thickness and Plaque Measured at the Second Survey
eTable 9. Associations of a *CETP* Genetic Score, Scaled to 10-mg/dL Higher HDL Cholesterol, With Occlusive CVD by Subgroups
eTable 10. Meta-analysis of rs2303790 With Coronary Heart Disease in the CKB and 2 Published Studies
eTable 11. Association of rs1333049 at the 9p21 Locus With Coronary Events in CKB
eTable 12. Comparison of the Associations of *CETP* Genetic Variants With Lipids in the CKB and the Global Lipids Genetics Consortium
eFigure 1. Study Participant Flowchart
eFigure 2. Associations of rs2303790 and a *CETP* Genetic Score With Lipoprotein Particle Size, Concentration, and Cholesterol Concentration
eFigure 3. Associations of a *CETP* Genetic Score With a Phenome-Wide Screen of 41 Disease Categories
eMethods 1. Details of Genotyping and Lipid and Lipoprotein Measurements
eMethods 2. Disease Outcomes and *ICD-10* Codes

This supplementary material has been provided by the authors to give readers additional information about their work.

© 2017 Millwood et al. *JAMA Cardiol*.

eTable 1. Allele Frequencies of 5 *CETP* Genetic Variants

	Chromosome	Position*	Reference allele	Alternate allele	Reference allele frequency	Alternate allele frequency
rs3764261	16	56993324	C	A	0.84	0.16
rs1800775	16	56995236	A	C	0.53	0.47
rs708272	16	56996288	G	A	0.59	0.41
rs9939224	16	57002732	G	T	0.88	0.12
rs2303790	16	57017292	A	G	0.98	0.02

*Build 37 (GRCh37.p13).

© 2017 Millwood et al. *JAMA Cardiol*.

eTable 2. Pairwise Linkage Disequilibrium* Among 5 *CETP* Genetic Variants

	rs3764261	rs1800775	rs708272	rs9939224	rs2303790
**rs3764261**		0.172	0.261	0.019	0.049
**rs1800775**	*0.999*		0.609	0.122	0.005
**rs708272**	*0.966*	*0.994*		0.082	0.011
**rs9939224**	*0.863*	*0.911*	*0.949*		0.002
**rs2303790**	*0.647*	*0.476*	*0.580*	*0.903*	

*Pairwise r^2 is shown above the shaded line and D' (italics) is shown below the shaded line.
Assessed among a subset of 66,513 individuals with first degree relationships excluded.

© 2017 Millwood et al. *JAMA Cardiol*.

eTable 3. Derivation of a *CETP* Genetic Score Weighted by Independent Associations With HDL Cholesterol

	Single variant model*					Multivariable model†				
	Effect/other allele‡	N	Per effect allele (mg/dL)	SE	P-value	Effect/other allele‡	N	Per effect allele (mg/dL)	SE	P-value
rs3764261	A/C	17,832	3.61	0.16	2.2×10^{-107}	A/C	17,761	2.74	0.19	1.9×10^{-45}
rs1800775	**A/C**	17,838	1.38	0.12	1.5×10^{-29}	**C/A**	17,761	0.66	0.19	7.6×10^{-04}
rs708272	A/G	17,826	1.95	0.12	2.9×10^{-56}	A/G	17,761	0.97	0.20	1.7×10^{-06}
rs9939224	G/T	17,838	2.32	0.19	1.3×10^{-34}	G/T	17,761	1.71	0.20	1.3×10^{-17}
rs2303790	G/A	17,835	6.06	0.42	9.4×10^{-47}	G/A	17,761	3.99	0.43	1.8×10^{-20}

*Adjusted for age and sex and stratified by area.

†Adjusted for age, sex and other *CETP* variants and stratified by area (further adjustment for hours since last meal, or CVD case-control status, made little difference to the results). The variance in HDL-cholesterol (r^2) explained by the model ranged from 0.031-0.055 across the ten areas.

‡The effect allele is defined as the allele associated with higher HDL-cholesterol. For rs1800775 in the single variant model the A allele was associated with higher HDL-cholesterol, but this changed to the C allele in the multivariable model. Conversion from conventional units to SI units: HDL-cholesterol from mg/dL to mmol/L multiply by 0.0259.

© 2017 Millwood et al. *JAMA Cardiol*.

eTable 4. Selected Baseline Characteristics Among Major Vascular Disease Cases and Common Vascular Disease Controls

Characteristic	Major vascular event cases*	Vascular disease controls†
No. of participants	24386	119724
Mean age (SD), years	58.9 (10.0)	50.4 (10.3)
Female, No. (%)	12480 (51.2)	71937 (60.1)
Urban, No. (%)	11066 (45.4)	49310 (41.2)
Education >6 years, No. (%)	4570 (18.7)	24249 (20.3)
Income >20,000 yuan/year, No. (%)	8320 (34.1)	49895 (41.7)
Prior disease history, No. (%)		
Coronary heart disease	1575 (6.5)	0.0 (0.0)
Stroke or transient ischaemic attack	1238 (5.1)	0.0 (0.0)
Diabetes	1841 (7.5)	2754 (2.3)
Hypertension	6576 (27.0)	10084 (8.4)
Medication use		
Anti-hypertensives	2889 (11.8)	3486 (2.9)
Statins	82 (0.3)	174 (0.1)
Regular smoker, No. (%)	7403 (30.4)	31346 (26.2)
Regular drinker, No. (%)	3806 (15.6)	18067 (15.1)
Mean physical activity (SD), MET-hours/day	15.5 (12.2)	22.1 (14.0)
Mean systolic blood pressure (SD), mmHg	145.2 (25.6)	129.7 (20.3)
Mean body mass index (SD), kg/m^2	24.1 (3.7)	23.6 (3.3)
Mean waist circumference (SD), cm	82.7 (10.3)	79.6 (9.6)
Mean random plasma glucose (SD), mg/dL	121.3 (59.8)	106.8 (39.1)

*Major vascular events (MVE) comprising myocardial infarction, coronary revascularisation, stroke or vascular death. Other vascular disease outcomes were a subset of MVE. †Common controls did not have a MVE, and excluded prior coronary heart disease or stroke or transient ischemic attack.

© 2017 Millwood et al. *JAMA Cardiol*.

eTable 5. Associations of *CETP* Genetic Variants With Lipids and Lipoproteins Measured by Clinical Biochemistry

	Effect/other allele*	N	Per effect allele (mg/dL)†	SE	P-value‡
**HDL-cholesterol**					
rs3764261	A/C	17,832	3.61	0.16	$2.2x10^{-107}$
rs1800775	A/C	17,838	1.38	0.12	$1.5x10^{-29}$
rs708272	A/G	17,826	1.95	0.12	$2.9x10^{-56}$
rs9939224	G/T	17,838	2.32	0.19	$1.3x10^{-34}$
rs2303790	G/A	17,835	6.06	0.42	$9.4x10^{-47}$
**LDL-cholesterol**					
rs3764261	A/C	17,832	0.94	0.40	0.018
rs1800775	A/C	17,838	0.62	0.29	0.033
rs708272	A/G	17,826	0.85	0.30	0.004
rs9939224	G/T	17,838	1.22	0.45	0.007
rs2303790	G/A	17,835	-0.49	1.01	0.630
**Total cholesterol**					
rs3764261	A/C	17,832	4.53	0.55	$2.8x10^{-16}$
rs1800775	A/C	17,838	2.02	0.41	$9.2x10^{-07}$
rs708272	A/G	17,826	2.69	0.41	$9.2x10^{-11}$
rs9939224	G/T	17,838	3.38	0.64	$1.0x10^{-07}$
rs2303790	G/A	17,835	5.77	1.42	$4.7x10^{-05}$
**Triglycerides**					
rs3764261	A/C	17,832	-4.85	2.04	0.017
rs1800775	A/C	17,838	-3.11	1.51	0.039
rs708272	A/G	17,826	-4.48	1.52	0.003
rs9939224	G/T	17,838	-3.03	2.33	0.194
rs2303790	G/A	17,835	-9.67	5.20	0.063
**Lipoprotein(a)**					
rs3764261	A/C	17,830	-0.03	0.02	0.109
rs1800775	A/C	17,836	-0.01	0.01	0.306
rs708272	A/G	17,824	0.01	0.01	0.617
rs9939224	G/T	17,836	-0.03	0.02	0.217
rs2303790	G/A	17,833	-0.12	0.05	0.016
**Apolipoprotein A1**					
rs3764261	A/C	17,789	4.64	0.32	$4.3x10^{-47}$
rs1800775	A/C	17,795	1.69	0.24	$1.5x10^{-12}$
rs708272	A/G	17,783	2.32	0.24	$5.6x10^{-22}$
rs9939224	G/T	17,795	2.95	0.37	$1.4x10^{-15}$
rs2303790	G/A	17,792	8.45	0.83	$1.3x10^{-24}$
**Apolipoprotein B**					
rs3764261	A/C	17,832	-0.01	0.31	0.977
rs1800775	A/C	17,838	0.13	0.23	0.579
rs708272	A/G	17,826	0.20	0.23	0.378
rs9939224	G/T	17,838	0.28	0.35	0.419
rs2303790	G/A	17,835	-1.46	0.78	0.062

*The effect allele is defined as the allele associated with higher HDL-cholesterol.
†Adjusted for age and sex and stratified by area (further adjustment for hours since last meal, or CVD case-control status, made little difference to the results). Conversion from conventional units to SI units: HDL- and LDL- and total cholesterol from mg/dL to mmol/L multiply by 0.0259; Lipoprotein(a) from mg/dL to µmol/L multiply by 0.0357; Triglycerides from mg/dL to mmol/L multiply by 0.0113; Apolipoproteins A1 and B from mg/dL to g/L multiply by 0.01.
‡P-values were unadjusted for multiple testing.

© 2017 Millwood et al. *JAMA Cardiol*.

eTable 6. Baseline Characteristics of the Study Population by rs2303790 Genotype and a *CETP* Genetic Score

	rs2303790*				*CETP* genetic score tertile*			
**Characteristic**	**AA**	**AG**	**GG**	**P-value†**	**Lower**	**Middle**	**Upper**	**P-value†**
No. of participants	144,595	6,525	74		50,345	53,483	47,219	
Mean age (SD), years	52.3 (10.8)	52.2 (10.8)	52.0 (10.8)	0.73	52.3 (10.8)	52.2 (10.8)	52.3 (10.8)	0.75
Female (%)	58.4	58.5	53.2	0.92	58.5	58.6	58.3	0.72
Urban (%)	41.8	44.7	51.5	1.5×10^{-6}	41.4	41.9	42.6	1.2×10^{-4}
Education >6 years (%)	19.8	21.0	22.5	0.04	19.7	19.9	20.1	0.19
Income >20,000 yuan/year (%)	40.3	40.1	45.1	0.92	40.2	40.3	40.4	0.51
Prior disease history (%)								
Hypertension	12.4	11.8	9.6	0.14	12.8	12.4	11.9	3.5×10^{-5}
Coronary heart disease	3.0	3.1	1.1	0.83	3.1	3.0	2.9	0.04
Stroke or transient ischaemic attack	1.6	1.5	2.4	0.87	1.6	1.6	1.5	0.05
Diabetes	3.4	3.2	6.3	0.47	3.6	3.3	3.3	0.04
Medication use (%)								
Anti-hypertensives	5.0	4.8	6.8	0.50	5.2	5.0	4.9	0.04
Statins	0.2	0.2	0.0	0.64	0.2	0.2	0.01	0.51
Regular smoker (%)	26.9	25.6	36.1	0.14	27.0	26.8	26.8	0.46
Regular drinker (%)	15.1	14.5	19.4	0.37	15.3	14.9	14.9	0.07
Mean physical activity (SD), MET-hours/day	20.7 (11.9)	20.7 (11.9)	20.9 (11.9)	0.90	20.8 (11.9)	20.7 (11.9)	20.8 (11.9)	0.92
Mean systolic blood pressure (SD), mmHg	132.6 (20.4)	132.2 (20.4)	128.6 (20.4)	0.08	132.7 (20.4)	132.5 (20.4)	132.4 (20.4)	0.02
Mean body mass index (SD), kg/m^2	23.6 (3.3)	23.6 (3.3)	23.4 (3.3)	0.93	23.6 (3.3)	23.7 (3.3)	23.6 (3.3)	0.40
Mean waist circumference (SD), cm	80.2 (9.3)	80.0 (9.3)	79.5 (9.3)	0.11	80.2 (9.3)	80.2 (9.3)	80.1 (9.3)	0.56
Mean random plasma glucose (SD), mg/dL‡	109.9 (43.2)	109.9 (43.2)	109.9 (43.2)	0.35	109.9 (43.2)	109.9 (43.2)	109.9 (43.2)	0.55

*Adjusted for age, sex and area.
†P for trend from a chi-squared for continuous traits and an analysis of variance for continuous traits.
‡Conversion from conventional units to SI units: multiply by 0.0555.

© 2017 Millwood et al. *JAMA Cardiol*.

eTable 7. Association of rs2303790 and a *CETP* Genetic Score, Both Scaled to 10-mg/dL Higher HDL Cholesterol, With LDL Cholesterol, According to Mean LDL Cholesterol in 10 Study Areas

				(a) rs2303790*		(b) *CETP* genetic score*	
**Study area** (R=rural, U=urban)	N	Mean LDL-cholesterol (mg/dL)†	SD	LDL-cholesterol effect (mg/dL)†	SE	LDL-cholesterol effect (mg/dL)†	SE
Gansu (R)	3,156	79.32	23.28	-2.17	3.63	-3.07	1.91
Sichuan (R)	1,577	87.43	29.32	-0.94	5.03	1.07	3.00
Zhejiang (R)	1,338	87.02	27.00	-3.03	5.19	0.87	2.98
Hunan (R)	3,401	89.48	27.77	-0.50	3.76	4.81	2.10
Suzhou (U)	715	89.49	29.09	4.37	7.59	-1.08	4.25
Henan (R)	3,071	90.70	24.69	-0.08	3.63	5.47	2.03
Harbin (U)	2,341	100.22	28.34	5.91	4.88	3.71	2.64
Liuzhou (U)	1,220	101.58	30.52	-6.14	5.73	1.12	3.54
Qingdao (U)	591	107.87	32.29	1.34	10.18	13.01	5.60
Haikou (U)	442	114.56	28.08	-15.68	11.95	-2.17	6.31

*Linear regression of mean LDL-cholesterol against LDL-cholesterol effect in each area: rs2303790 slope=0.13, P-value=0.44; *CETP* genetic score slope=-0.17, P-value=0.19.
†Adjusted for age and sex. Conversion from conventional units to SI units: LDL-cholesterol from mg/dL to mmol/L multiply by 0.0259.

© 2017 Millwood et al. *JAMA Cardiol*.

eTable 8. Associations of rs2303790 and a *CETP* Genetic Score, Both Scaled to 10-mg/dL Higher HDL Cholesterol, With Continuous Traits Measured at Baseline and Carotid Intima Media Thickness and Plaque Measured at the Second Survey

	**(a) rs2303790**				**(b) *CETP* genetic score**			
	N	Effect*	SE	*P*-value	N	Effect*	SE	*P*-value
**Baseline measurements**								
Systolic blood pressure (mm Hg)	151,194	-0.847	0.445	0.06	151,047	-0.737	0.254	0.004
Body mass index (kg/m^2)	151,193	0.000	0.070	1.00	151,046	-0.039	0.040	0.33
Waist circumference (cm)	151,194	-0.288	0.201	0.15	151,047	-0.126	0.115	0.27
Random plasma glucose (mg/dL)†	149,670	-0.458	0.885	0.61	149,524	-0.595	0.505	0.24
**Resurvey measurements**								
Carotid intima media thickness (mm)‡	22,273	0.008	0.009	0.37	22,241	0.007	0.005	0.17
Carotid plaque score§	22,274	0.064	0.059	0.28	22,242	0.048	0.033	0.15

*Adjusted for age and sex and stratified by area.

†Conversion from conventional units to SI units: multiply by 0.0555.

‡Mean taken from measurements of left and right carotid artery.

§Carotid plaque score derived from number and size of plaques in left and right carotid artery (Clarke et al, Eur J Prev Cardiol 2017 doi: 10.1177/2047487317689973).

© 2017 Millwood et al. *JAMA Cardiol*.

eTable 9. **Associations of a *CETP* Genetic Score, Scaled to 10-mg/dL Higher HDL Cholesterol, With Occlusive CVD by Subgroups**

	No. of cases	No. of controls	Odds ratio per 10 mg/dL higher HDL-cholesterol*	95% confidence interval	P-value†
Study area					
Rural	8,905	70,361	1.01	(0.91 - 1.12)	
Urban	9,645	49,283	0.96	(0.86 - 1.07)	0.51
Age (years)					
30-49	3,468	58,545	0.98	(0.84 - 1.15)	
50-59	5,746	36,730	1.04	(0.91 - 1.19)	
69-69	6,355	18,494	0.92	(0.80 - 1.05)	
70-79	2,981	5,875	1.01	(0.82 - 1.24)	0.61
Sex					
Male	8,903	47,746	0.96	(0.86 - 1.07)	
Female	9,647	71,898	1.01	(0.91 - 1.12)	0.54
Smoking					
Non-regular	13,087	88,322	0.97	(0.89 - 1.07)	
Regular	5,463	31,322	1.00	(0.87 - 1.15)	0.72
Alcohol drinking					
Non-regular	15,681	101,592	1.00	(0.92 - 1.08)	
Regular‡	2,829	17,543	0.92	(0.76 - 1.11)	0.44

*Adjusted for sex and age and stratified by study area, where appropriate.
†P for heterogeneity; obtained from Cochran's Q test.
‡Not all ten areas were included in the regression due to low numbers of cases in this subgroup.

© 2017 Millwood et al. *JAMA Cardiol*.

eTable 10. Meta-analysis of rs2303790 With Coronary Heart Disease in the CKB and 2 Published Studies

Study	N cases	N controls	Odds ratio (OR) per G allele	95% CI	P-value	G allele frequency cases	G allele frequency controls	LnOR	Var(LnOR)	Weight	Weight *LnOR
China Kadoorie Biobank	5,774	119,714	1.10	(0.97 - 1.25)	0.15	0.023	0.022	0.095	0.0044	228.12	21.74
Japan (Takeuchi 2012)*	4,399	7,672	0.83	(0.71 -0.97)	0.02	0.028	0.034	-0.186	0.0064	155.88	-29.04
Singapore (Cheng 2015)*	683	1,281	0.83	(0.54 – 1.27)	0.39	0.024	0.029	-0.186	0.0469	21.28	-3.97
**Overall**	**10856**	**128667**	**0.97**	**(0.88 – 1.07)**	**0.57**					**405.28**	**-11.27**

*Takeuchi et al 2012 PLoS One 7 e46385; Cheng et al 2015 Nat Commun 6 6063; SE and 95% CI are estimated based on the P-value reported. Cochrane Q-statistic for heterogeneity = 7.91; degrees of freedom=2; p=0.02.

© 2017 Millwood et al. *JAMA Cardiol*.

eTable 11. Association of rs1333049 at the 9p21 Locus with Coronary Events in CKB

Outcome	N cases	N controls	Odds ratio (OR) per C allele	95% CI	P-value
Major coronary events	5,774	119,659	1.08	(1.04-1.13)	$5.6x10^{-5}$
Myocardial infarction	3,122	119,659	1.10	(1.04-1.16)	$4.9x10^{-4}$

*Adjusted for sex and age and stratified by study area

© 2017 Millwood et al. *JAMA Cardiol*.

eTable 12. Comparison of the Associations of *CETP* Genetic Variants With Lipids in the CKB and the Global Lipids Genetics Consortium

		China Kadoorie Biobank (CKB): East Asian samples					Global Lipids Genetics Consortium (GLGC): European samples*					
	Effect/ other allele	EAF	N	Per allele effect (SD)[†]	SE	Effect P-value	EAF	N	Per allele effect (SD)	SE	Effect P-value	**Heterogeneity P-value[‡]**
**HDL-cholesterol**												
rs3764261	A/C	0.16	17,832	0.31	0.01	$2.2x10^{-107}$	0.29	177,533	0.241	0.004	$1x10^{-769}$	$6.8x10^{-7}$
rs1800775	A/C	0.53	17,838	0.12	0.01	$1.5x10^{-29}$	0.48	182,047	0.202	0.004	$<1x10^{-320}$	$4.1x10^{-13}$
rs708272[§]	A/G	0.41	17,826	0.17	0.01	$2.9x10^{-56}$	0.42	177,711	0.201	0.004	$<1x10^{-320}$	0.007
rs9939224	G/T	0.88	17,838	0.20	0.02	$1.3x10^{-34}$	0.76	92,820	0.249	0.007	$1.63x10^{-261}$	0.010
**LDL-cholesterol**												
rs3764261	A/C	0.16	17,832	0.03	0.01	0.018	0.29	164,865	-0.053	0.004	$2.22x10^{-34}$	$7.5x10^{-9}$
rs1800775	A/C	0.53	17,838	0.02	0.01	0.033	0.48	168,024	-0.041	0.004	$8.54x10^{-24}$	$2.0x10^{-8}$
rs708272[§]	A/G	0.41	17,826	0.03	0.01	0.004	0.42	163,717	-0.041	0.004	$4.09x10^{-25}$	$2.8x10^{-10}$
rs9939224	G/T	0.88	17,838	0.04	0.02	0.007	0.76	87,440	-0.046	0.008	$4.65x10^{-8}$	$7.4x10^{-7}$
**Total cholesterol**												
rs3764261	A/C	0.16	17,832	0.12	0.01	$2.8x10^{-16}$	0.29	177,497	0.050	0.004	$3.46x10^{-31}$	$5.9x10^{-6}$
rs1800775	A/C	0.53	17,838	0.05	0.01	$9.2x10^{-07}$	0.48	182,193	0.042	0.004	$2.10x10^{-28}$	0.365
rs708272[§]	A/G	0.41	17,826	0.07	0.01	$9.2x10^{-11}$	0.42	177,859	0.045	0.004	$2.04x10^{-29}$	0.026
rs9939224	G/T	0.88	17,838	0.09	0.02	$1.0x10^{-07}$	0.76	93,067	0.047	0.008	$2.53x10^{-11}$	0.026
**Triglycerides**												
rs3764261	A/C	0.16	17,832	-0.03	0.01	0.017	0.29	169,275	-0.040	0.004	$1.58x10^{-25}$	0.737
rs1800775	A/C	0.53	17,838	-0.02	0.01	0.039	0.48	172,715	-0.040	0.004	$1.33x10^{-26}$	0.120
rs708272[§]	A/G	0.41	17,826	-0.03	0.01	0.003	0.42	168,379	-0.036	0.004	$2.89x10^{-23}$	0.717
rs9939224	G/T	0.88	17,838	-0.02	0.02	0.194	0.76	89,485	-0.036	0.007	$7.39x10^{-7}$	0.423

*Willer et al Nat Genet 2013 45(11):1274-83; EAF obtained from 1KGP CEU.
[†]Adjusted for age and sex and stratified by area.
[‡]P for heterogeneity; obtained from Cochran's Q test.
[§]Proxy for rs708272 used from GLGC dataset: rs711752, pairwise linkage disequilibrium r^2=0.97.

© 2017 Millwood et al. *JAMA Cardiol*.

eFigure 1. Study participant flowchart

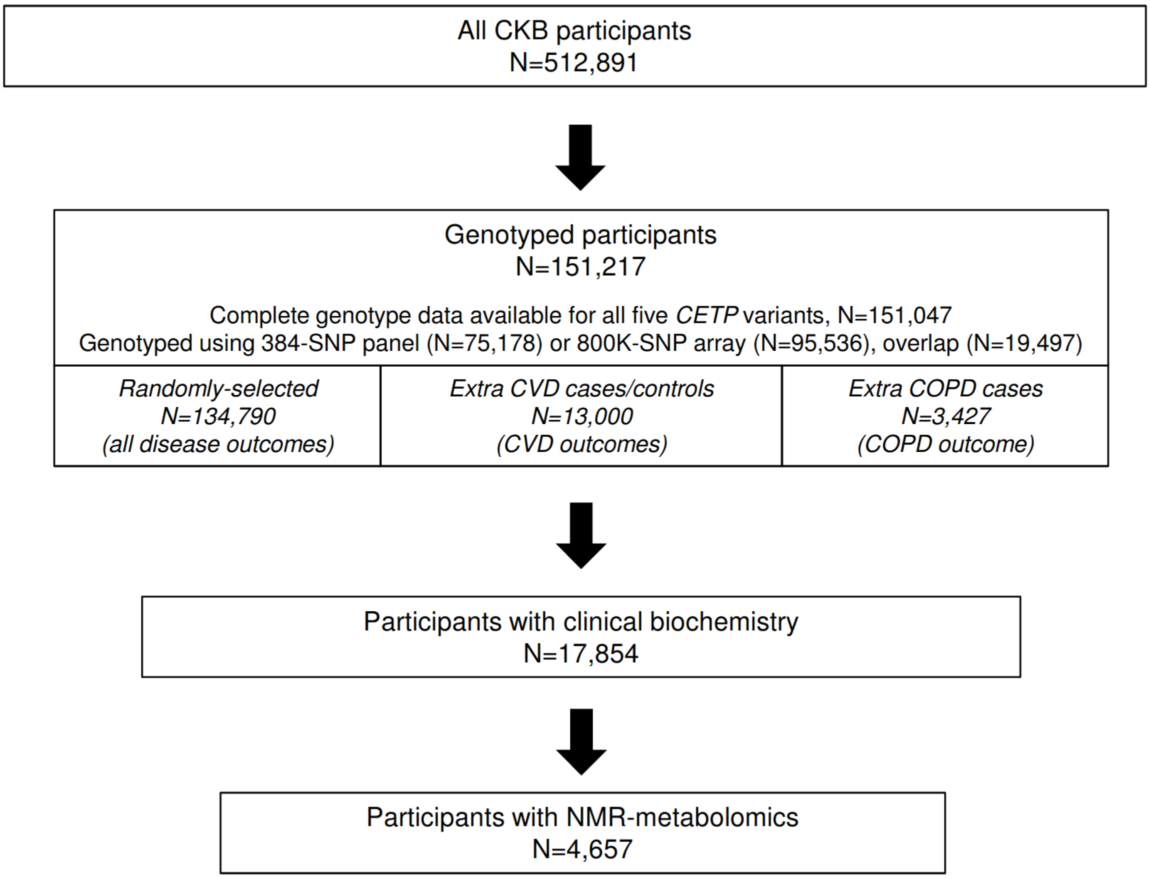

eFigure 1. Study participant flowchart. A total of 151,217 CKB participants were genotyped for five *CETP* variants. The majority of these (134,790) were randomly-selected and were used for analyses of all disease outcomes. Additional selected participants with CVD or COPD events and controls were used in analyses of relevant disease outcomes only. A subset of 17,854 genotyped participants underwent clinical biochemistry assays, and a further subset of 4,657 of these also had NMR-metabolomics assays performed.

© 2017 Millwood et al. *JAMA Cardiol*.

eFigure 2. Associations of rs2303790 and a *CETP* genetic score with lipoprotein particle size, concentration and cholesterol concentration

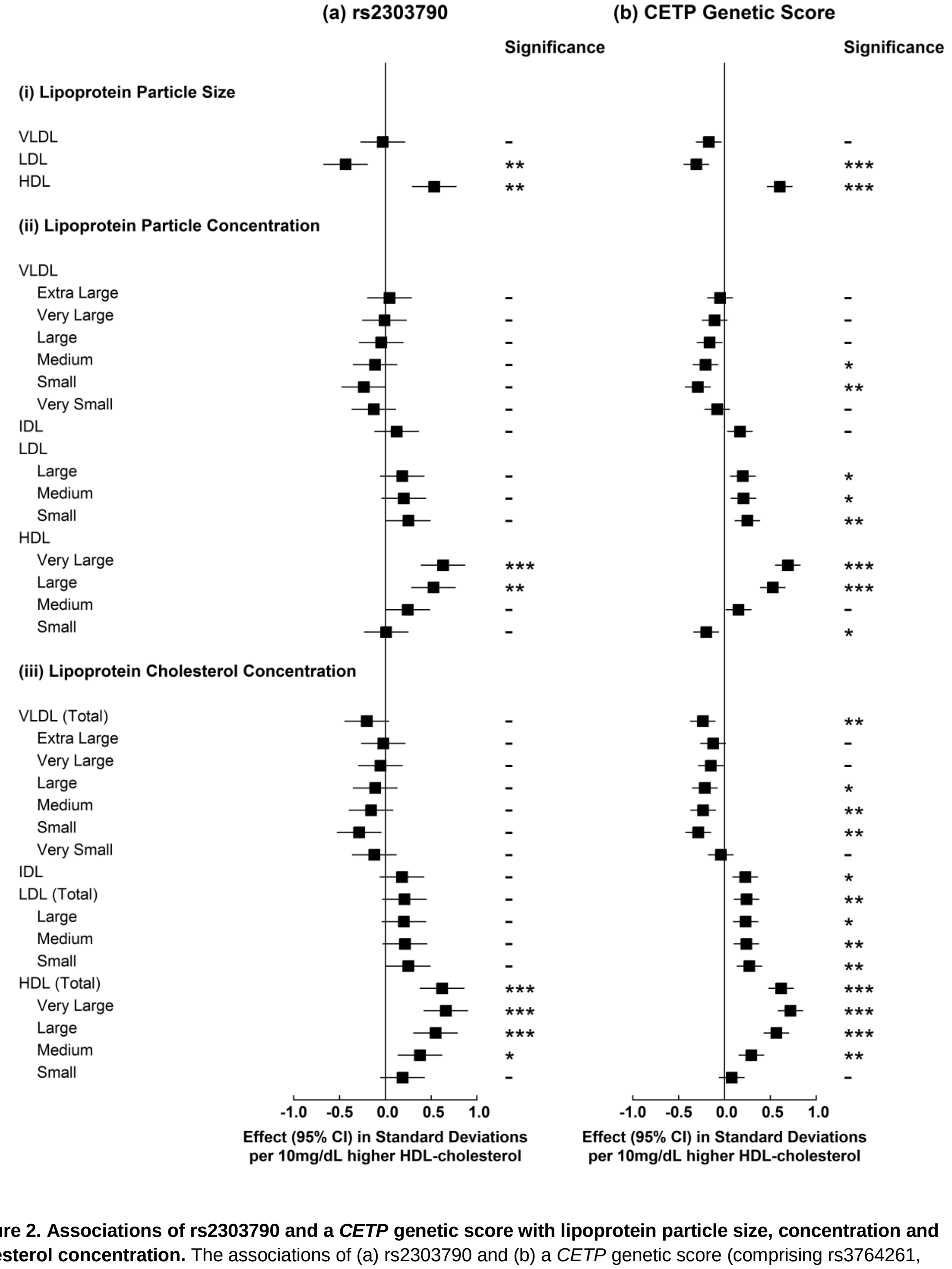

eFigure 2. Associations of rs2303790 and a *CETP* genetic score with lipoprotein particle size, concentration and cholesterol concentration. The associations of (a) rs2303790 and (b) a *CETP* genetic score (comprising rs3764261, rs1800775, rs708272, rs9939224, rs2303790), both scaled to 10 mg/dL higher HDL-cholesterol, with rank inverse normal transformation standardised traits measured by NMR-metabolomics in a subset of 4,657 individuals, were adjusted for sex and age and stratified by study area. Squares represent the effects in standard deviations of each trait. Horizontal lines represent the corresponding 95% confidence interval (CI). *P*-values after Bonerroni adjustment for the 18 principal components among the NMR traits are presented using the following notations: - >0.05; * < or = 0.05; ** < or = 0.01; *** < or = 0.0001.

© 2017 Millwood et al. *JAMA Cardiol*.

eFigure 3. Associations of a *CETP* genetic score with a phenome-wide screen of 41 disease categories

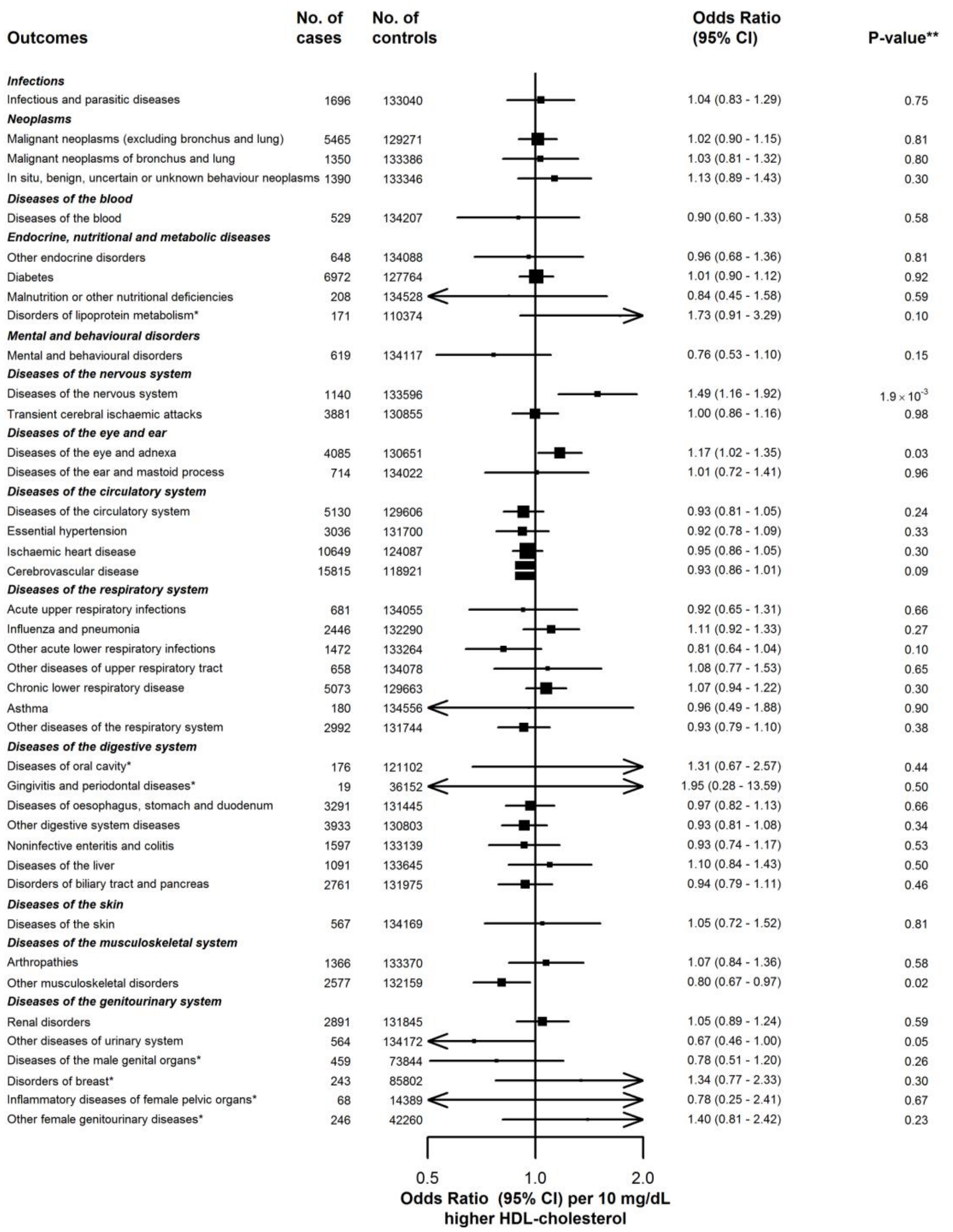

eFigure 3. Associations of a *CETP* genetic score with a phenome-wide screen of 41 disease categories. The associations of a *CETP* genetic score (comprising rs3764261, rs1800775, rs708272, rs9939224, rs2303790), scaled to 10 mg/dL higher HDL-cholesterol, with 41 ICD-10 coded disease categories, were adjusted for sex and age and stratified by study area. Squares represent the odds ratio (OR) with area inversely proportional to the variance of the log OR. Horizontal lines represent the corresponding 95% confidence interval (CI). *Not all ten areas contributed to the analyses for these outcomes due to sparse numbers of events. ***P*-values are unadjusted for multiple testing, but Bonerroni adjustment for 41 outcomes would result in a threshold of *P*<0.0012 (0.05/41).

© 2017 Millwood et al. *JAMA Cardiol*.

eMethods 1. Details of Genotyping and Lipid and Lipoprotein Measurements

Five *CETP* gene variants (rs3764261, rs1800775, rs708272, rs9939224, and rs2303790) were genotyped using a 384-SNP array (GoldenGate; Illumina) in 75,178 population-based (randomly-selected) individuals, and a custom-designed 800K-SNP array (Axiom; Affymetrix) in 95,536 individuals (79,725 of whom were population-based, and the remainder were selected specifically for nested case-control studies of CVD and COPD). There was overlap (n=19,497) between these datasets, resulting in *CETP* genotype data being available for a total of 151,217 individuals. Of these 151,217, 134,790 were population-based (included in analyses of all disease outcomes), 13,000 were selected CVD cases and controls (only included in analyses of CVD outcomes), and 3,427 were selected COPD cases (only included in analyses of COPD). The genotyping call rates were >99.97% for all five variants. Where genotype data were available from two sources, concordance was 99.9% for rs3764261 and rs1800775 and 100% for rs708272, rs9939224 and rs2303790. Where discordant, genotypes obtained from the Affymetrix Axiom® 800K-SNP array were used. Hardy-Weinberg equilibrium testing and linkage disequilibrium analyses were performed in a subset of participants from the 384-SNP Illumina® GoldenGate array with first-degree relationships removed (n= 66,513). Variants did not deviate from Hardy-Weinberg equilibrium, when stratified by region (P>10^{-3}).

A subset of the genotyped population (n=17,854; selected for CVD case-control studies) underwent clinical biochemistry assays at the CTSU laboratory, Oxford. Total cholesterol, LDL-cholesterol, HDL-cholesterol, triglycerides, lipoprotein(a), apolipoproteins B and A1 in EDTA plasma were assayed using Beckman-Coulter AU680 clinical chemistry analysers and manufacturers' reagents, calibrators and settings (Beckman-Coulter, UK), except LDL-cholesterol and HDL-cholesterol which used N-geneous reagents, calibrators and settings (Genzyme Diagnostics, UK) and lipoprotein(a) which used the Denka Seiken turbidimetric method with reagents and calibrators supplied by Randox Laboratories Limited, UK. The

© 2017 Millwood et al. *JAMA Cardiol*.

lipoprotein(a) method has documented traceability of lipoprotein(a) values to the WHO-IFCC Reference Material SRM2B for lipoprotein(a), achieved by direct comparison with the Northwest Lipid Metabolism and Diabetes Research Laboratories monoclonal antibody-based ELISA reference method. A further subset (n=4,657) of these 17,854 individuals also underwent metabolomics measurements through ^{1}H-NMR spectroscopy (providing data on 225 metabolic measures including lipid and lipoprotein particle profiles and ratios) at the Brainshake® Laboratory, University of Oulu.

© 2017 Millwood et al. *JAMA Cardiol*.

eMethods 2. Disease Outcomes and *ICD-10* Codes

Vascular outcomes

Major coronary events (MCE): Fatal IHD (ICD-10: I20-I25) or non-fatal myocardial infarction (ICD-10: I21) or coronary revascularisation procedures.

Fatal or non-fatal myocardial infarction (ICD-10: I21).

Fatal or non-fatal ischaemic stroke (ICD-10: I63).

Fatal or non-fatal intracerebral haemorrhage (ICD-10: 161).

Fatal or non-fatal stroke (ICD-10: I60, I61, 163, I64).

Occlusive CVD: Fatal IHD (ICD-10: I20-I25) or non-fatal myocardial infarction (ICD-10: I21) or coronary revascularisation procedures or fatal or non-fatal ischaemic stroke (ICD-10: I63).

Fatal cardiovascular disease (ICD-10: I00–99).

Major vascular events (MVE): Fatal or non-fatal myocardial infarction (ICD-10: I21) or coronary revascularisation procedures or fatal or non-fatal stroke (ICD-10: I60, I61, 163, I64) or fatal cardiovascular disease (ICD-10: I00–I99).

Common vascular controls: Exclude prevalent CHD, prevalent stroke or transient ischaemic attack or incident MVE.

Non-vascular outcomes

Diabetes (ICD-10: E10 – E14). Exclude prevalent diabetes from controls.

Chronic obstructive pulmonary disease (COPD) (ICD-10: J41 –J44). Exclude prevalent chronic emphysema/bronchitis from controls.

Chronic kidney disease (CKD) (ICD-10: E10.2+, E11.2+, E12.2+, E13.2+, E14.2+, I12.0, I12.9, I13.0, I13.1, I13.2, I13.9, M10.3, M32.1+, N02, N03, N04, N05, N08.3*, N11, N12, N13, N15, N18, N19, N25, N26, N27.1, N27.9, N28.9, O10.2, O10.3, R94.4, T86.1, Z94.0). Exclude prevalent kidney disease from controls.

Chronic liver disease including cirrhosis and hepatitis (ICD-10: CD-10 B18, B19, B25.1+, B58.1+, B94.2, K72, K73, K74, K75, K76, K77*, Z22.5) Exclude prevalent cirrhosis or hepatitis from controls.

Malignant neoplasms (ICD-10: C00-C97). Exclude prevalent cancer from controls.

Eye diseases (ICD-10: H00-H59).

Non-vascular mortality (all ICD-10 codes excluding I00-I99).

© 2017 Millwood et al. *JAMA Cardiol*.

Phenome-wide ICD-10 coded disease categories*

ICD-10 code start	ICD-10 code end	ICD-10 code start2	ICD-10 code end2	ICD-10 code start3	ICD-10 code end3	ICD-10 code start4	ICD-10 code end4	Endpoint description	Brief description
A00	B99							Certain infections and parasitic diseases	Infections
C00	C33	C37	C99					Malignant neoplasms (excluding bronchus and lung)	Malignant neoplasms excluding lung
C34	C34							Malignant neoplasms of bronchus and lung	Lung cancer
D00	D48							In situ, benign, uncertain or unknown behaviour neoplasms	Benign neoplasms
D50	D89							Diseases of the blood and blood-forming organs and certain disorders involving the immune mechanism	Haematological disorders
E00	E07	E15	E35					Disorders of thyroid gland, Other disorders of glucose regulation and pancreatic internal secretion, Disorders of other endocrine glands	Endocrine disorders excluding diabetes
E10	E14							Diabetes mellitus	Diabetes
E40	E77	E79	E90					Malnutrition, Other nutritional deficiencies, Obesity and other hyperalimentation, Metabolic disorders	Nutritional disorders excluding lipidaemias
E78	E78							Disorders of lipoprotein metabolism and other lipidaemias	Lipidaemias
F00	F99							Mental and behavioural disorders	Mental and behavioural disorders
G00	G44	G46	G99					Diseases of the nervous system (excluding TIA)	Nervous system disorders excluding TIA
G45	G45							Transient cerebral ischaemic attacks and related syndromes	Transient ischaemic attacks
H00	H59							Diseases of the eye and adnexa	Eye disorders
H60	H95							Diseases of the ear and mastoid process	Ear disorders
I00	I09	I11	I15	I26	I52	I70	I99	Diseases of the circulatory system (excluding hypertension, IHD, cerebrovascular disease)	Other vascular diseases
I10	I10							Essential (primary) hypertension	Hypertension
I20	I25							Ischaemic heart diseases	Ischaemic heart diseases
I60	I69							Cerebrovascular diseases	Cerebrovascular diseases
J00	J06							Acute upper respiratory infections	Acute upper respiratory infections
J09	J18							Influenza and pneumonia	Influenza/pneumonia
J20	J22							Other acute lower respiratory infections	Other acute lower respiratory infections
J30	J39							Other diseases of upper respiratory tract	Other upper respiratory tract diseases
J40	J44	J47	J47					Chronic lower respiratory diseases (excluding asthma)	Chronic lower respiratory diseases excluding asthma
J45	J46							Asthma, status asthmaticus	Asthma
J60	J99							Other diseases of the respiratory system	Other respiratory system diseases
J41	J44							Simple and mucopurulent chronic bronchitis, Unspecified chronic bronchitis, Emphysema, Other chronic obstructive pulmonary disease	COPD
K00	K04	K06	K14					Diseases of oral cavity, salivary glands and jaws	Oral cavity

© 2017 Millwood et al. *JAMA Cardiol*.

ICD-10 code start	ICD-10 code end	ICD-10 code start2	ICD-10 code end2	ICD-10 code start3	ICD-10 code end3	ICD-10 code start4	ICD-10 code end4	Endpoint description	Brief description
								(excluding gingivitis and periodontal diseases)	excluding periodontal
K05	K05							Gingivitis and periodontal diseases	Periodontal diseases
K20	K31							Diseases of oesophagus, stomach and duodenum	Stomach diseases
K35	K46	K55	K67	K90	K93			Diseases of appendix, Hernia, Other diseases of intestines, Diseases of peritoneum, Other diseases of the digestive system	Other digestive system diseases
K50	K52							Crohn disease [regional enteritis], Ulcerative colitis, Other noninfective gastroenteritis and colitis	Noninfective enteritis and colitis
K70	K77							Diseases of liver	Liver diseases
K80	K87							Disorders of gallbladder, biliary tract and pancreas	Gallbladder disorders
L00	L99							Diseases of the skin and subcutaneous tissue	Skin diseases
M00	M25							Arthropathies	Arthritis
M30	M99							Diseases of the musculoskeletal system and connective tissue (excluding arthropothies)	Other musculoskeletal disorders
N00	N29							Glomerular diseases, Renal tubulo-interstitial diseases, Renal failure, Urolithiasis, Other disorders of kidney and ureter	Renal disorders
N30	N39							Other diseases of urinary system	Urinary diseases
N40	N51							Diseases of male genital organs	Male genital diseases
N60	N64							Disorders of breast	Breast disorders
N70	N77							Inflammatory diseases of female pelvic organs	Female pelvic inflammatory diseases
N80	N99							Non-inflammatory disorders of female genital tract, Other disorders of the genitourinary system	Female other genitourinary diseases

*Codes outside the range A00-N99 were not considered in the current study. No exclusions for prevalent disease from the controls.

© 2017 Millwood et al. *JAMA Cardiol*.
